# Biomolecular Relationships Discovered from Biological Labyrinth and Lost in Ocean of Literature: Community Efforts Can Rescue Until Automated Artificial Intelligence Takes Over

**DOI:** 10.3389/fgene.2016.00046

**Published:** 2016-03-31

**Authors:** Rajinder Gupta, Shrikant S. Mantri

**Affiliations:** Computational Biology Lab, National Agri-Food Biotechnology InstituteMohali, India

**Keywords:** literature mining, named entity recognition, information retrieval, information extraction, community efforts, relationship database, interactions, relationships

Many brilliant minds are at work to decipher the biological labyrinth and as a result immense amount of information about biological entities and their relationships is getting accumulated in the form of published literature (Hunter and Cohen, 2006). To cater the needs of a researcher, many tools are designed to perform tasks of Named Entity Recognition (NER), Information Retrieval (IR), and Information Extraction (IE) viz. A Combined Clinical Concept Annotator (Kang et al., 2012), BANNER (Leaman and Gonzalez, 2008), Biblio-MetReS (Usie et al., 2014), BioTextQuest+ (Papanikolaou et al., 2014), BIOSMILE Web Search (Dai et al., 2008), E3Miner (Lee et al., 2008), EBIMed (Rebholz-Schuhmann et al., 2007), eFIP (Arighi et al., 2011), FACTA+ (Tsuruoka et al., 2008), GNSuite^1^, iHOP (Hoffmann and Valencia, 2004), MyMiner (Salgado et al., 2012), RLIMS-P(Hu et al., 2005), Anni (Jelier et al., 2008), CoPub (Frijters et al., 2008), MedScan (Novichkova et al., 2003), PPInterFinder (Raja et al., 2012), pGenN (Ding et al., 2015), SciMiner (Hur et al., 2009), BIGNER (Li et al., 2009), hybrid named entity tagger (Raja et al., 2014), and more such tools can be obtained from BIONLP resource^2^ and in detail analysis of many NLP tools is given by Krallinger et al. (2008) and Fleuren and Alkema (2015). Table 1 gives an informational and statistical insight into some of these literature mining tools, shedding light on their efficiency translated by statistical parameters viz. F-score, recall, and precision. Many tools are domain specific like kinase family specific but still calls for human intervention for exactitude and thus limit their usage. Moreover, the data output formats are sometimes too vague as name highlighting; to be put to use for bigger literature searches.

**Table 1:** **Informational (viz. data used, parameters for evaluation and working platform) and statistical (viz. ***f***-value, recall and precision) insights for a few literature mining tools with their brief description and links to the tools' home page**.

**Tool**	**Event/Data used**	**Parameters**	**Platform**	***F*-value (%)**	**Recall (%)**	**Precision (%)**	**Link**	**Description**
A Combined Clinical Concept Annotator (Kang et al., 2012)	i2b2 challenge	Concept exact match task	Web^**^	82.1	81.2	83.3	http://www.biosemantics.org/ACCCA_WEB	Concept annotation system for clinical records
Banner (Leaman and Gonzalez, 2008)	BioCreative 2 GM task	NER	Desktop	81.96	79.06	85.09	http://banner.sourceforge.net	Named entity recognition system, primarily intended for biomedical text
Biblio-MetReS (Usie et al., 2014)	Literature Databases and Journals	Biological entities and relationships	Desktop	37	27^*^	58	http://metres.udl.cat/	To reconstruct networks from an always up to date set of scientific documents
BIOSMILE Web Search (Dai et al., 2008)	BioCreAtIvE II GM tagging task and IAS task	NER and PPI article classifier	Web^**^	85.76	89.12	82.59	http://bws.iis.sinica.edu.tw/BWS/	Analyze articles for selected biomedical verbs and lists abstracts along with snippets by order of relevancy to protein–protein interaction
E3Miner (Lee et al., 2008)	100 random abstracts	E3 related data	Web	8^*^	74	97	http://e3miner.biopathway.org/e3miner.html	Extracts novel E3 discoveries and important findings related to specific E3s from the literature
RLIMS-P (Jelier et al., 2008)	BioCreative IV (BioCreative IAT)	Kinase, substrate and site	Web	92	96^*^	88	http://research.bioinformatics.udel.edu/rlimsp/	Rule-based text-mining program designed to extract protein phosphorylation information on protein kinase, substrate and phosphorylation sites from biomedical literature
Anni 2.0 (Frijters et al., 2008)	Micro-array data and multiple publications	Associations between biological entities	Web^**^	75.5^*^	76	75	http://biosemantics.org/anni/	Ontology-based interface to MEDLINE and retrieves documents and associations for several classes of biomedical concepts, including genes, drugs and diseases
PPInterFinder (Ding et al., 2015)	BioCreative workshop 2012	NER, IR	Web^**^	78.07	70.58	87.33	http://www.biominingbu.org/ppinterfinder/	Extracts human PPIs from biomedical literature using relation keyword co-occurrences with protein names to extract information on PPIs from MEDLINE abstracts
pGenN (Hur et al., 2009)	104 plant relevant abstracts	NER	Web	88.9	87.2	90.9	http://biotm.cis.udel.edu/gn/	A gene normalization tool for plant genes and proteins in scientific literature
SciMiner (Li et al., 2009)	BioCreAtIvE II	NER, IR	Desktop/Web	75.8	87.1	71.3	http://141.214.81.219/SciMiner/	Identifies genes and proteins using a context specific analysis of MEDLINE abstracts and full texts
BIGNER (Raja et al., 2014)	BioCreative 2 GM	NER	Web^**^	89.05	87.63	90.52	http://202.118.75.18:8080/bioner	To locate gene/protein names in biomedical literature

* *These values were self calculated from the given values*.

** *Out of order web-interfaces*.

*i2b2, Informatics for Integrating Biology and the Bedside; GM, Gene Mention; IAS, Interaction Article Sub-task; E3, ubiquitin-protein ligase; IAT, Interactive Task*.

The naming ambiguity in scientific literature is one of the major concerns for NER and sentence structure for IR and IE. Presently, NER tools need to maintain a comprehensive dictionary of all names, aliases and web-repository specific IDs or have their AI (Artificial Intelligence) defined algorithms trained on many test data sets. Many such dictionaries are available but the list is ever-increasing and so is the training data set. This results into investing more money, time and effort in obtaining a comprehensive list of names, aliases and IDs. A very comprehensive work on NLP can be found on BioNLP^3^. The availability of manpower or intellect is huge but there is acute scarcity of funds (Bourne et al., 2015), so we have to device optimized approaches to take care of the issues discussed in subsequent section.

## Issues in literature text mining

Let's have a deeper look into major concerns in biological literature mining:

- Non-standard naming conventions:
  The absence of any standard naming convention(s) for biological entities results in ambiguity and chaos. Presence of eponyms (Vedantam and Viswanathan, 2012) e.g., Bence Jones' protein, Wolfgram protein, Pokemon, Pikachurin etc., naming based on localization of proteins e.g., B-cell receptor-associated protein, naming based on function e.g. “101 kDa heat shock protein,” naming based on function and/or sequence similarity e.g., Epidermal growth factor-like protein 7 etc.; have all added to the complexity. A lot of research has been done to systematically name proteins and genes but no universal standards have been approved so far.
- Too many names:
  Owing to bad conventions followed to name biological entities many aliases (common name, acronym, descriptive name etc) for biological entities have come into existence (Iragne et al., 2004) e.g., 14-3-3 protein beta/alpha, Protein 1054, Protein kinase C inhibitor protein 1, KCIP-1 for one protein. Too many web-repositories have also resulted in many IDs for one entity e.g.,: P62258, P42655, CAB016200, CAB021109, CAB047350, HPA008445 for Uniprot Id P62258. And lastly non-uniform names e.g.,: AAD14 protein, AAD-14 protein, AAD 14 protein for Uniprot Id Q99415 adds to the problem.
- Defining relationships:
  English is the prime language of published research and it is evolving, and because of it NLP (Natural Language Processing) algorithms will never be 100% precise. Moreover, different people have different ways of putting up information and expressing their thoughts resulting in varied sentence corpuses making NER and IR tasks more difficult (Nadkarni et al., 2011). For defining relationships there are absolutely no conventions followed making it harder for the NLP tools.
- Scarcity of funds:
  The biological research demands too much of funds (Bourne et al., 2015) and more for its IT (Information Technology) support for the enormous amount of data that is generated. To provide a computational facility, that includes storage, data management, and making it available to the community through GUI (Graphical User Interface), it is an expensive affair. In addition, looking at amount of resources invested in devising NLP is too big to ignore.
- Unavailability of full text articles:
  Many high reputed journals provide their content for a price and only abstracts are available for free, making it harder for the researchers working in the domain to get hands on the missing information (Mower and Youngkin, 2008; Singh et al., 2011). There are ~3.7 million PMC full text articles and ~14 million Pubmed abstracts^4^, conveying we are only having ~25% of research at hand to go forward and this will increase further in days to come. Furthermore, the online unavailability of the supplementary material is a great setback for information extraction process (Evangelou et al., 2005).

## More data less information

NCBI^4^ houses 14,096,969 publications and a total of 64,815,068 genes and proteins; Uniprot^5^ houses 53,333,247 proteins collected from 1,007,941 publications. The data from Biogrid (Stark et al., 2006), one of the most extensive PPI repository has ~760 K interactions (I) for~80 K proteins (Pr) and covers~55 K publications (P) of total ~14 million present at Pubmed^4^. Some other PPI databases IntAct (Hermjakob et al., 2004; *P* = 13,892; Pr = 89,430; *I* = 564,831), DIP (Xenarios et al., 2000; *P* = 7,817; Pr = 28,215; *I* = 80,286), MINT (Zanzoni et al., 2002; *P* = 132,733; Pr = 35,553; *I* = 241,458), UniHI (Chaurasia et al., 2007; *E* = 22,300; *I* = 250,000), APID (Prieto and De Las Rivas, 2006; *P* = 416,124; Pr = 56,460; *I* = 322,579) also reflect the gap between the published literature and curated literature. No clear predictions can be made about how many interactions or relations we might be missing with such great amount of literature not being curated but surely a lot is missed. The gap will increase more and will become impassable if steps are not taken in time to bridge it.

The research also shows that so far we have been protein biased and all the relationship studies and repositories are dedicated to proteins (and on occasions, protein coding genes). We have totally missed the point that we are studying a system that comprises of rRNAs, ncRNAs, microsatellites, chemical components, drugs etc., and there is a crying need to bring them to the relationship databases too.

## Current progress

Many changes have been suggested and some have been implemented to take care of expanding biological literature and to make the information available as knowledge to the researchers in accessible formats or for computer programs to make sense of the text. Pubmed describes its own xml structure^6^ to store and provide the literature data. Such a structure having dedicated headers for the sections of the article are well suited for storing and retrieving of data but provide no assistance in making inferences form the text. Such an xml structure is limited in its usefulness to the NLP tools in just defining the sections such as title, abstract etc. that needs to be parsed.

More prominent work on making the structure of the format in which the literature is submitted has been carried out by Seringhaus and Gerstein (2007); suggesting to have a Structured Digital Abstract (SDA) and reporting of findings to appropriate databases, but community participation in populating databases/knowledgebases is very limited (Mazumder et al., 2010). SDA should be of great advantage to NLP and other computer programs to access the data (Superti-Furga et al., 2008) as it precisely defines the attributes such as species, gene, protein, mutation, interaction, experiment etc. in a well-organized and framed manner.

Winnenburg et al. (2008) proposed to have authors make annotations of their work and submit them according to some standard guidelines in addition to the original research paper. Shotton et al. (2009) also proposed many changes of which providing links to data from external sources; highlighting disease, organism, protein etc.; a document summary etc. are few important to take notice of. They also pressed for use of standard ontologies in biology literature. Clark et al. (2014) put forward an innovative approach to tackle the perishing literature issue by introduction of micropublications. They propose to have statement based models.

Ontologies also play a very important role in standardizing biological data such as classes of entities, relationships etc. (He and Xiang, 2013). Robinson and Bauer (2011) in their book have explained in depth about various aspects of bio-ontologies; data organization, integration, searching, computer reasoning etc. are few of them. The use of ontologies and their significance is well studied by Hur et al. (2011, 2015) in their work on gene-gene interactions and vaccines. Many more recommendations to improve the scientific literature's human and computer accessibility are available (Stevens et al., 2002; Leitner and Valencia, 2008; Sainani, 2008; Attwood et al., 2009; Fink et al., 2010) talking of liquid publications^7^ etc. are discussed in greater depths.

## The ways to pass the impassable

The scientific community has already spent jillions of money to uncover various biological phenomena, now to spend more to extract it from literature seems like a trivial task. Points enlisted below can help in addressing the concerns:

- Universal biomolecular entity and relationship database:
  A universal biomolecular relationships' database and an appropriate intuitive GUI needs to be designed and developed where researcher should submit their biomolecular relationship findings through an interactive data submission form. Every journal should encourage the authors to submit the data at this GUI in addition to submitting it to their journals and after the acceptance of the article the reported findings should go live. The database should house relationship data for all species and for all type of biomolecules in biological systems. All the entities of the database will be linked to external data sources to enhance the information of the entity, process etc. Inclusion of standard ontologies will further enrich the resource.
- New section to the e-version of publications/articles:
  A new section which defines the biomolecular entities and relationships in some standardized format should be added to the e-version of the publications/articles as described by many pioneers (Seringhaus and Gerstein, 2007; Clark et al., 2014). This way it should be easier for algorithm designers and developers to extract precise information from the published literature. The section can be in an XML (Extensible Markup Language) or OWL (Web Ontology Language) format (highly accepted across domains) that could be used by various tools and thus makes it easier to populate the relationship database. Journal editors need to take the big step and make it compulsory for the authors to add that new section.
- Data from existing relationship databases:
  Many relationship databases have manually curated relationship data (Xenarios et al., 2000; Hermjakob et al., 2004; Stark et al., 2006), that all can be added to the new repository and thus eliminating the need to redo the curation of the literature that has been done once or more. Using crawlers and APIs (Application Program Interface) that data should be integrated into the universal relationship database.
- Data backlog:
  Too much of literature is still lying in the dumps of data repositories viz. scientific journals that also need to be taken care of. We can start off with best of the tools (NER, IE, and IR) to handle them and over time let the community work on it to resolve clashes and normalize the relationships.

All the options should be used to eliminate the time gap between data availability i.e., publication of literature and its recognition in relevant databases for e.g., interaction databases, sequence database etc. The journals should provide programmatic access to their literature and supplementary data, allowing for speedy curation and fleeting integration in conformant databases. The authors from the journals open to such programmatic access will feel more to be a part of the knowledge evolution.

Currently the community efforts like Biocreative^8^ to solve the literature mining labyrinth have brought to life many new tools and approaches. Huang and Lu (2015) give in depth insights into the community programs and efforts. Similar initiatives need to be taken to accomplish this task also. The task is big but is a needed one, so we appeal the community to participate in designing the structure of form, new section in e-version etc.; developing standards for data submission, xml structure (as discussed by; Seringhaus and Gerstein, 2007), or some more ways like micropublications (Clark et al., 2014) etc.; and populating the databases with their research data and curating the data back log. Scientific journals need to make collaborative efforts to make it obligatory to submit literature in accordance with community established standards. Tools like PALM-IST (Mandloi and Chakrabarti, 2015) that use readily available relationship data from relationship databases to construct the biological interaction maps will be able to make good use of such relationship databases. Moreover, the precise relationship information will in turn provide diverse data sets for training our algorithms and should allow us to cover all literature that is not published with set norms. Semantics Scholar^9^, Plato^10^, and Aristo^11^ are artificial intelligence based natural language processing, visual knowledge extraction and reasoning systems, respectively, developed for searching relevant relationships from text, inferring information from images and answering questions from varied sources of information. Predictive potential of such novel tools in the field of biology should improve drastically by utilizing community cumulated biomolecular relationship knowledge.

Automating the literature mining process using NER, IE and IR has proved to be a costly affair with slow progress as compared to the speed of new research getting published. More robust approaches need to be thought of to accommodate the gap between the published literature and manually curated literature. One way to achieve this is by having a universal biomolecular relationship database and data submission GUI where all biological relationship information is shared by the authors themselves. Extensive community efforts will be required to achieve such an enormous task.

## Author contributions

SM: Conceived and designed the study. RG and SM: Performed literature review, wrote the manuscript and approve this final draft.

## Funding

This work was financially supported by the core grant of National Agri-Food Biotechnology Institute (NABI), Mohali, India and the Department of Biotechnology, Government of India.

### Conflict of interest statement

The authors declare that the research was conducted in the absence of any commercial or financial relationships that could be construed as a potential conflict of interest.

## Footnotes

^1^https://www.idi.ntnu.no/~satre/biocreative/IAT/.

^2^http://zope.bioinfo.cnio.es/bionlp_tools/.

^3^http://bionlp.org/.

^4^ftp://ftp.ncbi.nlm.nih.gov/pubmed/.

^5^http://www.uniprot.org/downloads.

^6^https://www.nlm.nih.gov/bsd/licensee/elements_descriptions.html.

^7^Casati, F.; Giunchiglia, F.; Marchese, M. Liquid Publications, Scientific Publications Meet the Web; Technical Rep. DIT-07-073, Informatica e Telecomunicazioni; University of Trento: Trento, Italy, 2007. http://eprints.biblio.unitn.it/1313/1/073.pdf.

^8^http://www.biocreative.org/.

^9^http://allenai.org/semantic-scholar.html.

^10^http://allenai.org/plato.html.

^11^http://allenai.org/aristo.html.
